# First draft genome assembly of the desert locust, *Schistocerca gregaria*

**DOI:** 10.12688/f1000research.25148.2

**Published:** 2021-05-21

**Authors:** Heleen Verlinden, Lieven Sterck, Jia Li, Zhen Li, Anna Yssel, Yannick Gansemans, Rik Verdonck, Michiel Holtof, Hojun Song, Spencer T. Behmer, Gregory A. Sword, Tom Matheson, Swidbert R. Ott, Dieter Deforce, Filip Van Nieuwerburgh, Yves Van de Peer, Jozef Vanden Broeck

**Affiliations:** 1 Laboratory of Molecular Developmental Physiology and Signal Transduction, KU Leuven, Leuven, 3000, Belgium; 2 Laboratory of Bioinformatics and Evolutionary Genomics, Ghent University, Ghent, 9000, Belgium; 3 Center for Plant Systems Biology, Ghent University - VIB, Ghent, 9052, Belgium; 4 Department of Biochemistry, Genetics and Microbiology, University of Pretoria, Pretoria, 0002, South Africa; 5 Laboratory of Pharmaceutical Biotechnology, Ghent University, Ghent, 9000, Belgium; 6 NXTGNT, Ghent University, Ghent, 9000, Belgium; 7 Station d' Ecologie Théorique et Expérimentale, UMR 5321 CNRS et Université Paul Sabatier, Moulis, 09200, France; 8 Department of Entomology, Texas A&M University, College Station, Texas, TX 77843-2475, USA; 9 Department of Neuroscience, Psychology and Behaviour, University of Leicester, Leicester, LE1 7RH, UK

**Keywords:** Eco-devo, large genome size, locust plague, Orthoptera, pest insect, phenotypic plasticity, polyphenism, swarm

## Abstract

**Background**: At the time of publication, the most devastating desert locust crisis in decades is affecting East Africa, the Arabian Peninsula and South-West Asia. The situation is extremely alarming in East Africa, where Kenya, Ethiopia and Somalia face an unprecedented threat to food security and livelihoods. Most of the time, however, locusts do not occur in swarms, but live as relatively harmless solitary insects. The phenotypically distinct solitarious and gregarious locust phases differ markedly in many aspects of behaviour, physiology and morphology, making them an excellent model to study how environmental factors shape behaviour and development. A better understanding of the extreme phenotypic plasticity in desert locusts will offer new, more environmentally sustainable ways of fighting devastating swarms.

**Methods**: High molecular weight DNA derived from two adult males was used for Mate Pair and Paired End Illumina sequencing and PacBio sequencing. A reliable reference genome of *Schistocerca gregaria* was assembled using the ABySS pipeline, scaffolding was improved using LINKS.

**Results**: In total, 1,316 Gb Illumina reads and 112 Gb PacBio reads were produced and assembled. The resulting draft genome consists of 8,817,834,205 bp organised in 955,015 scaffolds with an N50 of 157,705 bp, making the desert locust genome the largest insect genome sequenced and assembled to date. In total, 18,815 protein-encoding genes are predicted in the desert locust genome, of which 13,646 (72.53%) obtained at least one functional assignment based on similarity to known proteins.

**Conclusions**: The desert locust genome data will contribute greatly to studies of phenotypic plasticity, physiology, neurobiology, molecular ecology, evolutionary genetics and comparative genomics, and will promote the desert locust’s use as a model system. The data will also facilitate the development of novel, more sustainable strategies for preventing or combating swarms of these infamous insects.

## Introduction

**Figure 1.**
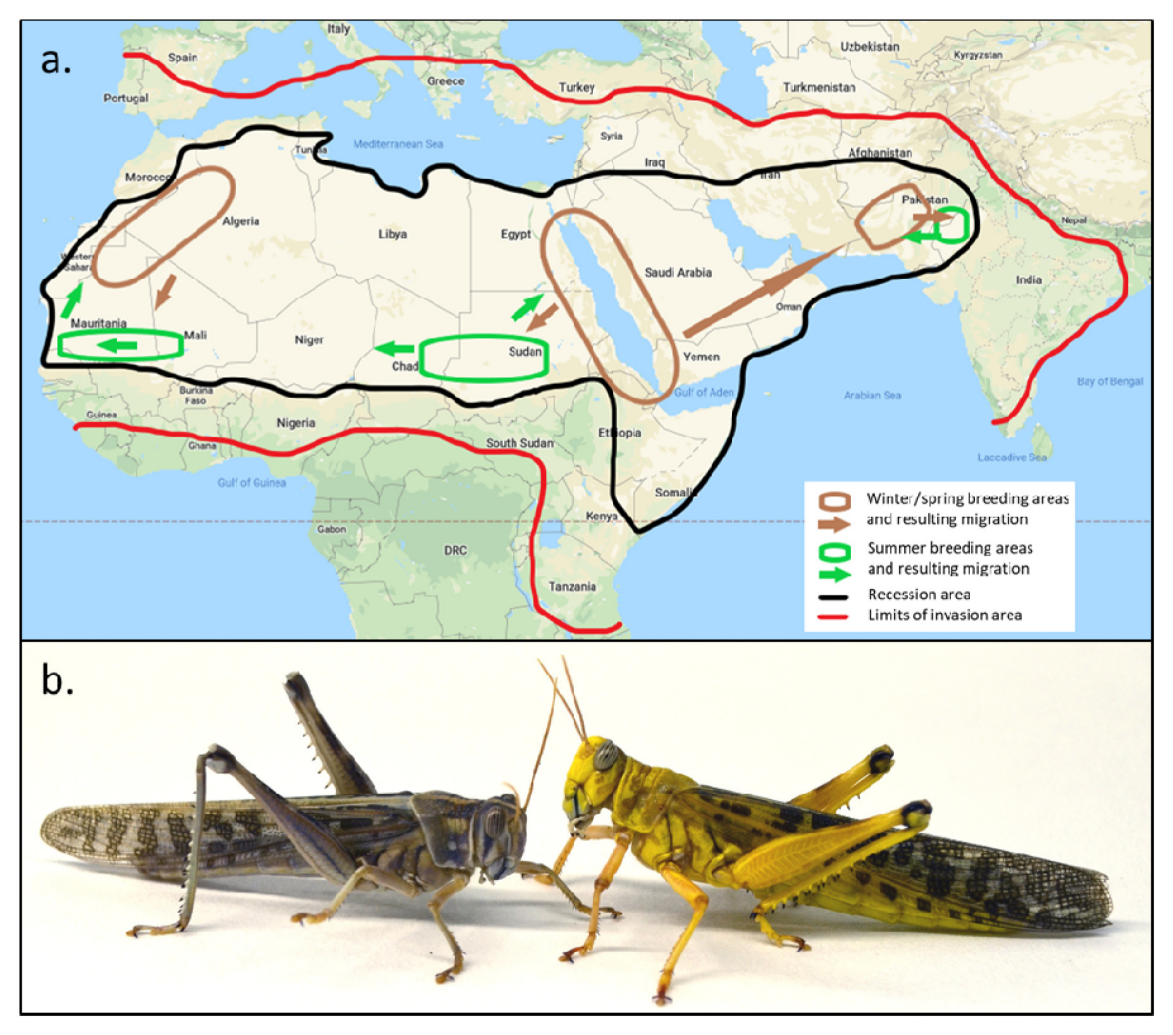
Geographical distribution of the desert locust and a picture of two adult male desert locusts, one in the solitarious phase and the other in the gregarious phase. ( **a**) Geographic distribution of the desert locust. During ‘recession’ periods, desert locusts are restricted to the semi-arid and arid regions of Africa, the Arabian Peninsula and South-West Asia that receive less than 200 mm of annual rain. The recession area covers about 16 million km ^2^ in 30 countries. Within this recession area, locusts move seasonally between winter/spring and summer breeding areas. During outbreaks, desert locusts may spill into more fertile adjacent regions, threatening an area of some 29 million km ^2^ comprising 60 countries as outbreaks escalate into upsurges and further into plagues. The recession breeding areas and migration patterns may have predictive value to understand how the swarms will migrate Range of the non-swarming southern sub-species *S. gregaria flaviventris* not shown. Figure based on information from FAO Locust Watch ( Cressman, 2016; Symmons & Cressman, 2001), map derived from Google Map Data ©2020 Google. ( **b**) Phase polyphenism in desert locusts, using the example of sexually mature males. The gregarious male (right) is brightly coloured, while the solitarious male relies on camouflage colours. In this staged scene, the solitarious male was forced into close proximity of the gregarious male and is seen retreating from its conspecific. Photo by H. Verlinden and R. Verdonck.

Locust plagues have been recorded since Pharaonic times in ancient Egypt. In the Bible ( *Exodus 10*), locust swarms are described as one of the major destructive plagues and still today they form a serious threat to crops and food security of over 60 countries across more than 20% of the world’s total land surface ( Figure 1a). Swarms can cover areas up to several hundred square kilometres and migrate up to 200 km per day. Per square kilometre, a swarm that contains about 40 million locusts can eat the same amount of food in one day as about 35,000 people. The damage done by a locust plague is on the same level as a major drought (FAO Locust Watch; De Vreyer *et al.*, 2012). The long-term socio-economic impact of these swarms is significant. The loss of harvest is disastrous for local farmers and leads to towering local food prices, also affecting non-farming families. The poorest households are often hit the hardest. Malnourishment of children and expecting mothers endangers their long-term health and growth. School enrolment rate fell by a quarter during plagues in 1987–89 in Mali, with girls being particularly affected ( Courcoux, 2012). Human activities in turn affect the propensity of locusts to swarm through factors such as land use (e.g. agriculture, wood extraction, urbanization), political relations between affected countries and the effects of climate change (FAO Locust Watch, http://www.fao.org/ag/locusts/en/info/info/index.html; Cullen *et al.*, 2017; Meynard *et al.*, 2020).

Desert locusts ( *Schistocerca gregaria* Forskål) are grasshoppers (Orthoptera: Acrididae) that exhibit ‘phase polyphenism’, an extreme form of phenotypic plasticity that evolved as an adaptation to the drastic changes that can occur in their environment. Locusts can develop into two extremely divergent, population density-dependent phenotypes, which are tailored to very different ecological requirements. Under low population densities, locusts appear in the solitarious phase and live a solitary life in which they avoid each other. In periods with abundant rainfall, rapid vegetation growth creates a favourable habitat that permits large increases in local population sizes. However, when food becomes scarce again, solitarious locusts are forced to aggregate on the remaining plants. This crowding causes the transformation into the swarming gregarious phase *,* beginning with rapid changes in behaviour that include a switch to increased locomotion and mutual attraction. The prolonged crowding drives slower but equally profound changes in colouration, morphology ( Figure 1b) and physiology. Compounded across multiple generations, locust populations can aggregate further into huge, ruinous swarms capable of crossing continents and oceans in search of food. Populations may crash in the absence of sufficient resources or following human intervention, leading once more to scattered low density solitarious phase populations. The transition between locust phases is thus reversible and occurs gradually through the expression of intermediate phenotypic states ( Cullen *et al.*, 2017; Pener & Simpson, 2009; Symmons & Cressman, 2001; Verlinden *et al.*, 2009).

Orthoptera (grasshoppers, crickets and allies) belong to the Polyneoptera, a clade that represents one of the major lineages of winged insects (Pterygota) and comprises around 40,000 known species and ten orders of hemimetabolous insects ( Misof *et al.*, 2014; Wipfler *et al.*, 2019). Other major neopteran (Pterygota that can flex their wings over their abdomen) lineages are Acercaria (mostly sucking insects such as lice or true bugs) and Holometabola (insects with complete metamorphosis). At present, only 25 sequenced polyneopteran genomes are reported on NCBI and i5k ( http://i5k.github.io/arthropod_genomes_at_ncbi), unequally distributed over five different orders ( *Extended data*, Supplementary Table S1 ( Verlinden *et al*., 2020)). When including *S. gregaria*, the genomes of five orthopteran species, representing five different subfamilies, are now available. In addition to representing a paradigmatic example of phenotypic plasticity, the desert locust is an important research model for generating advances in a wide variety of fundamental and applied scientific areas, including biomechanics, ecology, pest control, neurobiology and physiology. For instance, the relatively large body size of locusts has been instrumental in discovery of a multitude of insect neuropeptides ( Schoofs *et al.*, 1997). Moreover, the globally increasing interest in the use of insects as food or feed also applies to the desert locust, which is a highly nutrient-rich, edible insect that is gaining much attention as a potential, climate-friendly food source ( van Huis *et al.*, 2013).

The devastating socio-economic impact of locust swarms, together with the opportunity this species offers to investigate the phenotypic interface of molecular processes and environmental cues highlight the importance of sequencing the desert locust genome. However, the extremely large estimated genome size of 8.55 Gb ( Camacho *et al.*, 2015; Fox, 1970; John & Hewitt, 1966; Wilmore & Brown, 1975) predicted a formidable challenge. Moreover, previous transcriptomics and chromosome size data from the desert locust ( Badisco *et al.*, 2011; Camacho *et al.*, 2015), as well as comparisons with the genome of the distantly related migratory locust, *Locusta migratoria* (6.5 Gb; Wang *et al.*, 2014), suggested that the non-coding part and repetitive regions of the desert locust genome might be greatly expanded as compared to other insect genomes, presenting additional challenges to sequencing and assembly ( Dominguez Del Angel *et al.*, 2018; Tørresen *et al.*, 2019). Our team has overcome these hurdles and presents here the ~8.8 Gb genome of the desert locust assembled from short Illumina Mate Pair (MP) and Paired End (PE) reads and long PacBio reads. This new genomic resource, the largest insect genome yet sequenced and assembled, will complement decades of research on this species, enhancing the desert locust’s role as an important comparative model system. The genome will permit exciting new opportunities to examine mechanisms of phenotypic plasticity, social behaviour, physiological and morphological specialization. Moreover, it will open up new avenues to find better ways of fighting the notorious swarms they can cause. The desert locust genome will also enable better understanding of genome size evolution and the early phylogeny of winged insects.

## Methods

### Sequencing strategy

A hybrid sequencing approach was adopted consisting of both Illumina short read sequencing to get sufficient coverage for accurate contig assembly, and complementary PacBio long read sequencing to allow efficient scaffolding of the contig assembly. The Illumina and first PacBio sequencing were performed on high-molecular-weight DNA derived from the central nervous system (central brain, optic lobes, ventral nerve cord), fat body and testes of one adult male inbred for seven generations. A second round of PacBio sequencing used DNA from another male from the same lineage, with two additional generations of inbreeding (for details on the animal material and genomic DNA extraction, see *Extended data*, Supplementary Methods ( Verlinden *et al.*, 2020)).

### Illumina sequencing

The concentration of the *S. gregaria* high molecular weight DNA sample was measured with PicoGreen (Invitrogen) fluorimetry, after which DNA integrity was confirmed by gel electrophoresis (1% E-Gel; Invitrogen). The sample was divided for Illumina MP and PE sequencing library preparation.

The MP sequencing library was prepared from 1 µg of the sample with a “Nextera Mate Pair Library prep kit” (Illumina). The PE library was prepared with a “NEBNext Ultra II library prep kit” (NEB) from 2 µg of the sample, sheared to 500 bp fragments using an S2 focused-ultrasonicator (Covaris). Size selection (600–700 bp) was performed for both libraries in a 2% E-Gel (Invitrogen). The quality of the libraries was confirmed with a Bioanalyzer High Sensitivity DNA Kit (Agilent). The MP and PE libraries were quantified by qPCR, according to Illumina's “Sequencing Library qPCR Quantification protocol guide” (version February 2011) and pooled at a molar ratio of 25% MP – 75% PE for sequencing on Hiseq3000 (2 × 150 cycles, 16 lanes; Illumina).

### PacBio sequencing

The library preparation for PacBio sequencing was performed with a "SMRTbell Template Prep Kit 1.0" according to the PacBio protocol (version 100-286-000). For each of the two libraries, 10 µg of the *S. gregaria* high-molecular-weight DNA was used as input in two parallel 50-µl reactions.

For library size selection, a "0.75% Dye-Free Agarose Gel Cassette” (ref: BLF7510) was used on a Blue Pippin (Sage Science) with the "0.75% DF Marker S1 high-pass 15–20kb" protocol for a lower cut-off of 12 kb. Fragment size distribution was determined with a “DNA 12000 kit” (ref: 5067-1508) for the first library and a “Fragment Analyzer (Agilent) - High Sensitivity Large Fragment 50 kb kit” (ref: DNF-464-0500) for the second library. The resulting libraries had an average length of 16.5 and 22 kb, respectively.

No extension time was used for the sequencing as recommended for size selected libraries in the “Quick Reference Card 101-461-600 version 07”. The first run was performed on a PacBio RSII System (V4.0 chemistry, polymerase P6). Fifteen additional runs were performed on a PacBio Sequel system with 2.0 Chemistry, polymerase and SMRTCells. The same conditions were used to sequence 20 more SMRTCells with the second library on the PacBio Sequel system.

### Genome assembly

PE short read data were pre-processed with bbduk v38.20 from the BBTools package to remove adapters and low-quality reads. Illumina MP read data were cleaned and separated into true MP data and likely MP data in nxTrim ( O’Connell *et al.*, 2015). The long-read PacBio data were pre-processed using CANU v1.7 ( Koren *et al.*, 2017) to obtain trimmed and corrected reads. Cleaned short-read PE and MP data were then assembled using the ABySS v2.1.1 pipeline ( Simpson *et al.*, 2009) up to scaffold stage, using a k-mer value of 120. Parameters for ABySS were optimized away from default values to achieve better performance (for all parameter settings see *Extended data*, Supplementary Table S2 ( Verlinden *et al*., 2020)). The assembly was further improved by using the PacBio data as input for LINKS ( Warren *et al.*, 2015).

### Annotation of repetitive elements and noncoding RNAs

Two strategies were used to identify and annotate repetitive elements. First, *de novo* annotation was carried out by RepeatModeler v2.0 and LTR_FINDER v1.0.7 ( Xu & Wang, 2007) to build a custom repeat library. Second, a homology-based approach was used to search for repetitive elements in the assembled genome using the repetitive element library of RepeatMasker v4.1.0 and RepeatProteinMask v4.1.0. The results of both strategies were combined into a non-redundant set of repetitive elements. Subsequently, the library was used to mask repetitive elements by employing RepeatMasker v4.1.0 ( Tarailo-Graovac & Chen, 2009).

Transfer RNAs (tRNAs) were predicted by tRNAscan-SE v1.31 ( Lowe & Eddy, 1997) with default parameters. To predict non-coding RNAs (ncRNAs), such as microRNAs (miRNAs), small nuclear RNAs (snRNAs), and ribosomal RNAs (rRNAs), the desert locust genome was screened against the RNA families (Rfam) v14.1 database ( Griffiths-Jones *et al.*, 2003) by the cmscan program of Infernal v1.1.2 ( Nawrocki & Eddy, 2013). To supplement our predictions of miRNAs, miRNA sequences from the *L. migratoria* genome ( Wang *et al.*, 2015) were extracted and searched in the *S. gregaria* genome by *BLASTN* with options “-task blastn-short -ungapped -penalty -1 -reward 1” ( Camacho *et al.*, 2008). The alignment result was filtered using a mismatch cutoff of 3 bp. Specifically, the stem-loop structure of each potential miRNA was predicted by miRNAFold ( Tav *et al.*, 2016) using each alignment with 110 bp upstream and downstream sequences. Then the RNAfold program of ViennaRNA v2.4.14 ( Lorenz *et al.*, 2011) was used to calculate the minimum free energy (MFE) of each stem-loop structure. If a potential miRNA had several predicted stem-loop structures, the one with the minimum MFE was selected as representative. Putative miRNAs located within protein coding sequences or repetitive elements were discarded. Finally, the results based on Rfam and the migratory locust genome were combined into a non-redundant prediction of miRNAs.

### Gene prediction and functional annotation

Protein-coding genes in the desert locust genome were predicted using three approaches. (1) RNA-Seq reads (see *Extended data*, Supplementary Methods ( Verlinden *et al*., 2020)) were mapped to the desert locust genome using HISAT2 v2.1.0 ( Kim *et al.*, 2015) with parameter “--max-intronlen” set to 1,000,000 to increase the maximum allowed intron length during read mapping. Then, StringTie v2.1.1 ( Pertea *et al.*, 2015) was used to assemble potential transcripts based on RNA-Seq alignments to the desert locust genome. Subsequently, TransDecoder v5.0.2 was used to identify open reading frames (ORFs) within the assembled transcripts which resulted in 20,201 ORFs with start and/or stop codons. We also built *de novo* assembled transcripts based on the pooled RNA-Seq reads of all samples with Trinity v2.8.4 ( Grabherr *et al.*, 2011; Haas *et al.*, 2013) and obtained 285,499 transcripts (including isoforms), of which 57,870 putative protein-coding transcripts and 305 rRNA candidates were identified by Trinotate v3.1.1 ( Bryant *et al.*, 2017). This was complemented with 34,974 ESTs of the desert locust from NCBI ( Badisco *et al.*, 2011). The assembled transcripts and ESTs were then aligned to the desert locust genome with Program to Assemble Spliced Alignments (PASA v2.4.1) ( Haas *et al.*, 2003). (2) For *ab initio* gene prediction, we used a hard-masked genome in which genomic repetitive elements were substituted by ‘N’. To build a training set for the *ab initio* gene predictors, we extracted 498 complete genes with both start and stop codons from the 500 longest ORFs predicted by TransDecoder, based on the above RNA-Seq analysis with HISAT2 and StringTie. Augustus v3.3.3 ( Stanke *et al.*, 2006) SNAP v2006-07-28 ( Korf, 2004) and GlimmerHMM v3.0.4 ( Majoros *et al.*, 2004) were trained on this training set and then used to predict potential gene models. Furthermore, combined with the RNA-Seq alignments, BRAKER2 v2.1.5 ( Hoff *et al.*, 2019) was used to predict protein-coding genes based on the above-mentioned training model of Augustus. (3) The proteomes of the migratory locust, *Locusta migratoria* ( Wang *et al.*, 2014); the African malaria mosquito, *Anopheles gambiae*; the domestic silk moth, *Bombyx mori*; the fruit fly, *Drosophila melanogaster*; the kissing bug, *Rhodnius prolixus*; the red imported fire ant, *Solenopsis invicta*; the red flour beetle, *Tribolium castaneum*; and the Nevada dampwood termite, *Zootermopsis nevadensis* from Ensembl Metazoa (release-47), as well as the proteins in UniRef100 (release-2020_01) for the clade Polyneoptera (Taxonomy ID: 33341) were used to assist gene predictions with homologous proteins. Exonerate v2.4.0 ( Slater & Birney, 2005) was used to perform spliced alignments of the proteins with the maximum intron length set to 1 Mb. To integrate the predictions from all three gene-prediction approaches, EvidenceModeler v1.1.1 ( Haas *et al.*, 2008) was used to produce a non-redundant gene set. Functional annotation of the predicted protein-coding genes was done by running BlastP ( Altschul *et al.*, 1990) using an e-value cut-off of 1×10 ^-5^ against the public protein databases Uniprot/SwissProt ( Magrane, 2011; The UniProt Consortium, 2019) and NCBI NR (RefSeq non-redundant protein record). Protein family (Pfam) domain information and Gene Ontology (GO) terms were added using InterProscan ( Mitchell *et al.*, 2019).

## Results and discussion

### Genome size and assembly

Initial input data for the assembly comprised (i) 1,316 Gb of Illumina short read data, of which 1,009 Gb remained after cleaning and trimming, and (ii) 112 Gb of long reads from PacBio sequencing. The resulting assembly, using the ABySS pipeline, consisted of 8.5 Gb in ~1.6 M contigs with an N50 of 12,027 bp. Scaffolding with the MP data using ABySS resulted in 8.6 Gb in 1.2 M scaffolds with an N50 of 66,194 bp. The PacBio data as input for LINKS further improved the scaffolded assembly derived from ABySS, doubling the N50 and maximum length and reducing the number of sequences by half. The final assembly consists of 8,817,834,205 bp organised in 955,015 scaffolds with an N50 of 157,705 bp ( Table 1).

**Table 1.** Results of the assembly for the desert locust genome.

	Total	Total size (bp)	N50 (bp)	N90 (bp)	Largest (bp)	Mean length (bp)
Contigs	1,648,200	8,561,922,307	12,027	5,375	202,979	5,194.71
Scaffolds (MP)	1,233,802	8,632,364,377	66,194	15,575	1,561,787	8,350.11
Scaffolds (PacBio)	955,015	8,817,834,205	157,705	29,453	3,339,430	9,233.20

Scaffolds (MP), Scaffolds reached with the Mate Pair data using the ABySS pipeline; Scaffolds (PacBio), improved scaffolds with the PacBio data as input for LINKS; N50, the sequence length of the shortest contig/scaffold at 50% of the total genome length; N90, the sequence length of the shortest contig/scaffold at 90% of the total genome length

### Repetitive elements and noncoding RNAs

In total, repetitive elements account for 62.55% of the desert locust genome ( Table 2), which is more than the 58.86% repetitive elements in the published migratory locust genome ( Wang *et al.*, 2014). Screening the desert locust genome against the Rfam v14.1 database identified 121,581 tRNAs, 1,302 rRNAs, 121 miRNAs, and 361 snRNAs ( *Extended data*, Supplementary Table S3 ( Verlinden *et al.*, 2020)).

**Table 2.** Repetitive elements in the genomes of the desert locust, *Schistocerca gregaria*, and the migratory locust, *Locusta migratoria* ( Wang *et al*., 2014).

	*Schistocerca gregaria*		*Locusta migratoria*	
Repeat Types	Length (bp)	P%	Length (bp)	P%
DNA	2,390,333,660	27.1	1,480,538,225	22.69
LINE	2,438,094,307	27.6	1,332,720,207	20.42
SINE	28,032,199	0.32	141,176,698	2.16
LTR	637,406,118	7.23	508,675,263	7.80
Other	165	0.00	32,017	0.00
Unknown	871,233,596	9.88	406,097,360	6.22
Total	5,515,243,572	62.55	3,840,808,141	58.86

DNA, DNA transposons; LINE, long interspersed nuclear element retrotransposon; SINE, short interspersed nuclear element retrotransposon; LTR, long terminal repeat retrotransposon; Other, repeats classified to other than the above mentioned types; Unknown, repeats that cannot be classified; P%, percentage of the genome.

In addition to the 121 evolutionary conserved miRNAs identified from Rfam, blasting with miRNAs previously identified in the migratory locust (from small RNA sequencing-based and homology-based approaches; Wang *et al.*, 2015) identified a further 686 miRNAs in the desert locust genome, resulting in a total of 807 identified miRNAs ( *Extended data*, Supplementary Table S3 ( Verlinden *et al.*, 2020)). Of these 807 miRNAs, 676 are located on short scaffolds without any protein-coding gene. Among the 121 miRNAs identified based on Rfam, 81 have no homologs in the migratory locust genome.

### Protein-coding genes

**Figure 2.**
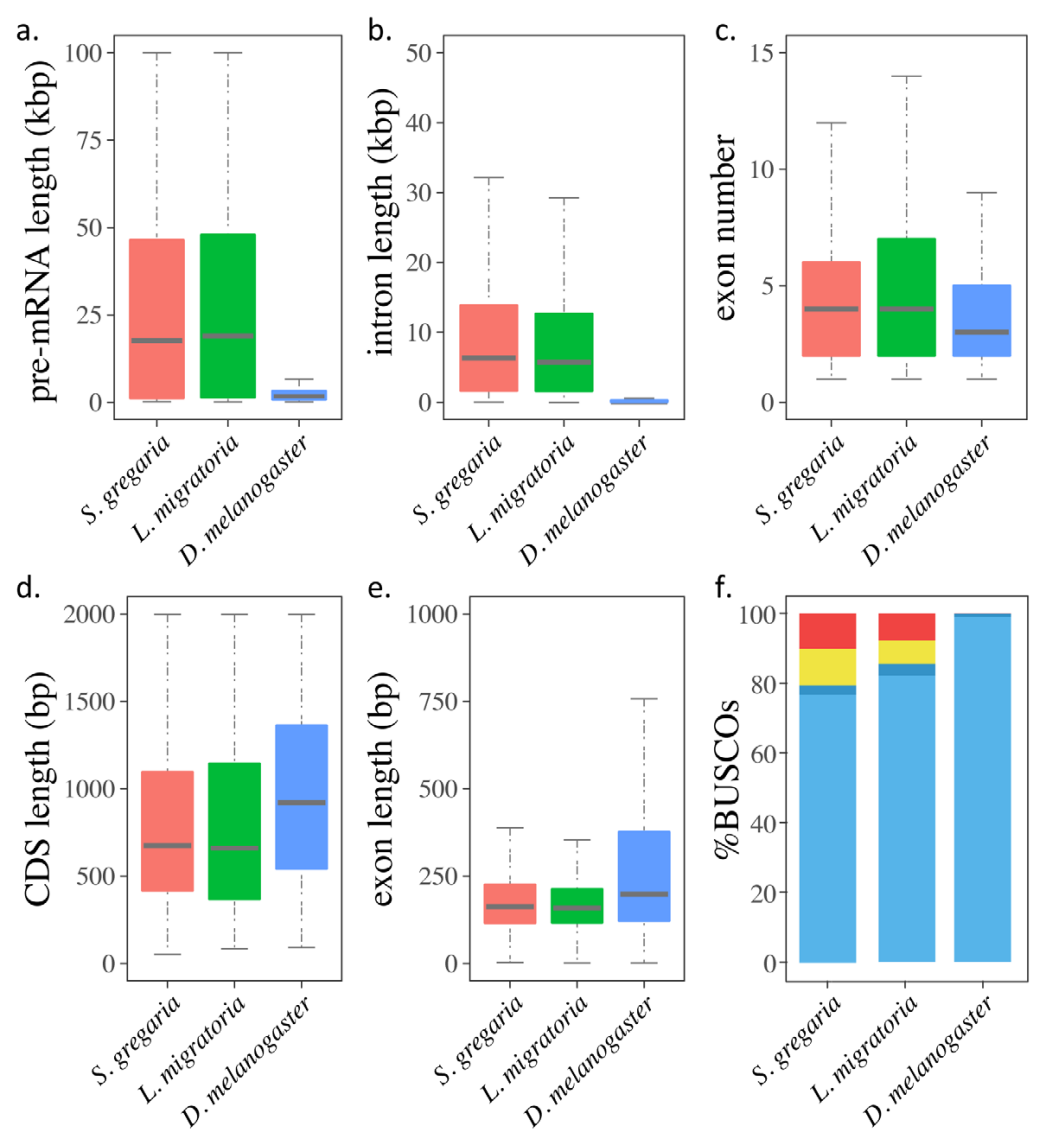
Gene characteristics and BUSCO assessment in the genomes of the desert locust, *Schistocerca gregaria*, the migratory locust, *Locusta migratoria* ( Wang *et al.*, 2014) and the fruit fly, *Drosophila melanogaster* ( Adams *et al.*, 2000). ( **a**– **e**) Boxplots of ( **a**) pre-mRNA lengths; ( **b**) intron lengths; ( **c**) exon numbers; ( **d**) coding sequence (CDS) lengths; and ( **e**) exon lengths in the three genomes. ( **f**) BUSCO assessments of the gene sets in the three genomes. The stacked bars indicate the percentages of genes that are complete (light blue), duplicated (dark blue), fragmental (yellow) and missed (red).

In total, 18,815 protein-encoding genes are predicted in the desert locust genome ( *Extended data*, Supplementary Table S4 ( Verlinden *et al.*, 2020)). The average pre-mRNA length is 54,426 bp, with an average coding sequence (CDS) length of 1,137 bp and an average intron length of 12,522 bp, values which are comparable to those of the published migratory locust genome ( Wang *et al.*, 2014). Although both locust genomes have longer pre-mRNAs with bigger introns and more exons than the *Drosophila melanogaster* genome ( Adams *et al.*, 2000), their average CDS and exon length are in fact shorter ( Figure 2 and Table 3). The BUSCO assessment of the current gene set (protein mode) shows that it includes 79.4% complete genes in the insecta_odb10 dataset ( Simão *et al.*, 2015), which closely matches the result from the BUSCO genome completeness assessment (genome mode) of 80.9% ( *Extended data*, Supplementary Table S5 ( Verlinden *et al*., 2020)). Comparing the BUSCO assessment of the Trinity assembly (91.2% completeness) with that of the current gene set of the genome indicates that the present genome assembly is still missing genes that are present in the transcriptomes. The BUSCO assessment of the predicted genes in the desert locust genome shows fewer complete genes than for the published *Locusta migratoria* and *Drosophila melanogaster* genomes ( Figure 2). Among the 18,815 predicted genes in the desert locust genome, 13,646 (72.53%) obtained at least one functional assignment based on similarity to known proteins in the databases. Pfam domain information could be added to 10,395 (55.25%) predicted genes, and 6,470 (34.39%) predicted genes could be assigned a GO term ( *Extended data*, Supplementary Table S6 ( Verlinden *et al.*, 2020)).

**Table 3.** Summary statistics on gene information for the desert locust, *Schistocerca gregaria*, and the migratory locust, *Locusta migratoria* ( Wang *et al*., 2014).

	*Schistocerca gregaria*	*Locusta migratoria*
Genome		
Size (bp)	8,817,834,205	6,524,990,357
Scaffold N50 (bp)	157,705	322,700
GC content	0.406	0.407
Gene		
Total gene number	18,815	17,307
Average pre-mRNA Length (bp)	54,426	54,341
Average CDS length (bp)	1,137	1,160
Average intron length (bp)	12,522	11,159
Average exon length (bp)	216	201
Average exon number per gene	5.26	5.77

Scaffold N50, the sequence length of the shortest scaffold at 50% of the total genome length; CDS, coding sequence.

## Conclusions

Here, we present the first draft genome sequence of the desert locust, *Schistocerca gregaria*, a swarming pest species with significant socio-economic and ecological impact. With the current locust crisis in mind, it should be clear that despite ongoing monitoring and control operations, we are still in urgent need of more locust research to foster development of effective management strategies. Sequencing and assembling the desert locust genome has been both challenging and ground-breaking due to the enormous size of the genome and its extremely large proportion of repetitive elements. The desert locust genome is the largest insect genome sequenced and assembled to date. As is the case for the second and third largest assembled insect genomes, the expanded genome size is caused by accumulation of repetitive regions and intron elongation ( *Locusta migratoria*, 6.5 Gb; Wang *et al.*, 2014; *Clitarchus hookeri*, 4.2 Gb; Wu *et al.*, 2017). Sequencing the desert locust genome is an important step to advance our knowledge of these animals. It will enable future studies to examine the very complex relationship between environmental cues and phenotypic plasticity, and in particular the question of how this is regulated at the molecular level. A better understanding of the desert locust’s molecular biology will facilitate the development of novel, more sustainable strategies for controlling these pests.

## Data availability

### Underlying data

European Nucleotide Archive: First draft genome of Schistocerca gregaria, a swarm forming grasshopper species. Accession number PRJEB38779; https://identifiers.org/ena.embl:PRJEB38779.

This accession contains all genome and transcriptome data. The annotations are also available via the ORCAE platform ( https://bioinformatics.psb.ugent.be/orcae/overview/Schgr).

### Extended data

Figshare: First draft genome assembly of the desert locust, Schistocerca gregaria - extended data. https://doi.org/10.6084/m9.figshare.12654026.v2 ( Verlinden *et al.*, 2020).

This project contains the following extended data:

- Supplementary Methods (DOCX). Containing details of Animal material, Genomic DNA extraction, Library construction, sequencing for RNA-Seq and *de novo* transcriptome assembly.
- Supplementary Table S1 (DOCX). Available Polyneopteran genomes (incl. *Schistocerca gregaria* for comparison).
- Supplementary Table S2 (DOCX). Software parameter settings.
- Supplementary Table S3 (DOCX). Transfer RNA (tRNA), microRNA (miRNA), small nuclear RNA (snRNA) and ribosomal RNA (rRNA) content of the desert locust genome.
- Supplementary Table S4 (DOCX). Desert locust genome annotation details.
- Supplementary Table S5 (DOCX). BUSCO assessments for the genomes of the desert locust, Schistocerca gregaria, and the migratory locust, Locusta migratoria ( Wang *et al.*, 2014).
- Supplementary Table S6 (DOCX). Functional annotation of the proteome of the desert locust.

Extended data are available under the terms of the Creative Commons Attribution 4.0 International license (CC-BY 4.0).
