# Supplementary material for: First draft genome assembly of the desert locust, Schistocerca gregaria
Source: F1000Res. 2021 May 21;9:775. Originally published 2020 Jul 27. [Version 2] doi: 10.12688/f1000research.25148.2 (PMC7607483; doi:10.12688/f1000research.25148.2)
Supplement: Peer review history

# Peer review: Reviewer response for version 2

**DOI:** 10.5256/f1000research.56735.r85888

**Published:** 2021-06-14

**Authors:** Surya Saha

**Affiliations:** 1 Boyce Thompson Institute for Plant Research, Ithaca, NY, USA

The authors have responded satisfactorily to my comments. I look forward to future work from the authors that address the additional analysis I had mentioned in my first report.

Is the work clearly and accurately presented and does it cite the current literature?

Yes

If applicable, is the statistical analysis and its interpretation appropriate?

Not applicable

Are all the source data underlying the results available to ensure full reproducibility?

Yes

Is the study design appropriate and is the work technically sound?

Yes

Are the conclusions drawn adequately supported by the results?

Yes

Are sufficient details of methods and analysis provided to allow replication by others?

Yes

Reviewer Expertise:

Arthropod genomics and transcriptomics, Comparative genomics and Metagenomics

I confirm that I have read this submission and believe that I have an appropriate level of expertise to confirm that it is of an acceptable scientific standard.

# Peer review: Reviewer response for version 2

**DOI:** 10.5256/f1000research.56735.r85889

**Published:** 2021-05-24

**Authors:** Uwe Homberg

**Affiliations:** 1 Animal Physiology, Department of Biology, Center for Mind, Brain and Behavior (CMBB), University of Marburg, Giessen, Germany

This paper looks fine now.

Is the work clearly and accurately presented and does it cite the current literature?

Yes

If applicable, is the statistical analysis and its interpretation appropriate?

Yes

Are all the source data underlying the results available to ensure full reproducibility?

Yes

Is the study design appropriate and is the work technically sound?

Yes

Are the conclusions drawn adequately supported by the results?

Yes

Are sufficient details of methods and analysis provided to allow replication by others?

Yes

Reviewer Expertise:

neurobiology of the desert locust, neuropeptide research in desert locust, neuroanatomy of the locust brain

I confirm that I have read this submission and believe that I have an appropriate level of expertise to confirm that it is of an acceptable scientific standard.

# Peer review: Reviewer response for version 1

**DOI:** 10.5256/f1000research.27753.r72658

**Published:** 2020-11-02

**Authors:** Joshua B. Benoit

**Affiliations:** 1 Department of Biological Sciences, University of Cincinnati, Cincinnati, OH, USA

This is an extremely important agricultural pest and having a genome for this species will allow for more future comparisons among locust species. This study represents a great deal of work and the techniques used are appropriate and well described. There is some room for improvement, but a valuable contribution. 

1. I would suggest to add a little more biological interpretation. Was there anything of interest and unique identified? Specifically, anything related to the transition from solitary to swarming.
2. The assembly is of sufficient quality for some comparisons to other insects, but there might be issues with the low BUSCO score. This was similar to the Locusta genome.  Please check the BUSCO score of the Trinity assembly to determine if the missing genes are present. If the missing genes are present in the de novo assembly,  I would make sure to make the de novo assembly available until a higher quality genome is available. 
3. Were any bacterial symbiont present or microbial contamination detected? How were these accounted for in the assembly?
4. As a future goal, I would suggest adding techniques for chromosome scaffolding (e.g. Hi-C). This genome is fine as a draft, a higher quality version will be needed for future comparisons. 

Is the work clearly and accurately presented and does it cite the current literature?

Yes

If applicable, is the statistical analysis and its interpretation appropriate?

Yes

Are all the source data underlying the results available to ensure full reproducibility?

Yes

Is the study design appropriate and is the work technically sound?

Yes

Are the conclusions drawn adequately supported by the results?

Yes

Are sufficient details of methods and analysis provided to allow replication by others?

Yes

Reviewer Expertise:

Insect physiology, molecular biology, and genomics

I confirm that I have read this submission and believe that I have an appropriate level of expertise to confirm that it is of an acceptable scientific standard.

# Response

**Published:** 2021-05-06

**Authors:** Heleen Verlinden

**Affiliations:** Catholic University of Leuven, Belgium

We would like to thank the reviewer for this positive feedback and for recognising the challenges we have faced. Since sequencing technologies are evolving very fast, we agree that future work will further improve the current genome assembly. This is why we designated this as the “first genome assembly”. In addition, with the release of our genome data, we are convinced that many additional analyses are becoming possible. Locusts have always been -and still are- research models for studying a wide range of research questions in different areas of interest. With this manuscript, we therefore chose not to focus on a very specific research question, but communicate the main results obtained of our genome sequencing effort. Moreover, given the current locust situation, we felt that it was important to make available this first genome draft to the international scientific community and to stimulate readers -worldwide- to address their specific research question(s).

 The BUSCO assessment on the Trinity assembly shows that it includes 91.2% complete genes in insecta_odb10 dataset. Compared with the BUSCO values (genomic 80.9% vs geneset 79.4%) of our current genome assembly this indeed indicates that our genome assembly still misses some BUSCO genes that are present in our transcript databases. It proves that our current genome assembly can still be further improved, as we are of course aware. The Trinity assembly is included in the gene set we made available.

 As already clarified in our answer to a very similar comment made by reviewer 2, we took many precautions to avoid contaminations and we did not observe any. This is now specified in the supplementary materials and methods.

 We fully agree with the reviewer that additional techniques may further improve this draft genome assembly.

# Peer review: Reviewer response for version 1

**DOI:** 10.5256/f1000research.27753.r72656

**Published:** 2020-10-27

**Authors:** Surya Saha

**Affiliations:** 1 Boyce Thompson Institute for Plant Research, Ithaca, NY, USA

This work is timely given the locust outbreaks in East Africa and recently in parts of West Asia with the devasting impact on crops of small holder farmers in these regions besides secondary impacts on nutrition and human health. The large genome size and repetitive regions make this a challenging genome to assemble. The phase polyphenism of the gregarious and solitarious adults make this a fascinating system to study for social behavior and physiology in arthropods. A high-quality chromosomal length genome assembly for S. gregaria will lay the foundation for genetics and phenotyping of this important insect pest. The methods for the genome assembly, protein coding and non-coding gene annotation are clearly described in the paper and in the extended data. Inclusion of the parameters used is helpful for the reproducibility of the genome assembly process. I commend the authors on a well written manuscript.

 Although this is a valuable contribution to Polyneoptera genomics, it is possible to do a better job of utilizing the new genome and annotation for comparison with other sequenced relatives in Polyneoptera, especially the migratory locust.  In my humble opinion, the manuscript can be improved a lot if these issues are addressed.

 1. This manuscript can become a tour de force for locust genomics if additional analysis and discussion were to be included. Gene families related to energy consumption and detoxification already identified in the migratory locust are of particular interest. There are two other aspects that, if addressed, will be of value to the community.

 1a.The authors mention a greater presence of ncRNA elements in the S. gregaria genome. The association of these potential regulatory elements with protein coding genes based on RNA data from this paper and other public data will be useful.

 1b. The other point is about a more detailed characterization of the repeat elements that account for 62% of the genome. A GenomeScope or similar plot of the heterozygosity in the Illumina reads might be useful to understand the repetitive structure.

 I know this adds additional burden on the authors but I hope they see my rationale.

 2. Endosymbionts been reported for other locust genomes (https://www.mdpi.com/2075-4450/11/10/655 ^1^). These are typical by products of insect genome assembly. Were any microbial contigs found in the assembly for known endosymbionts?

 I had a few minor points:

 1. The introduction states that the potentially expanded non-coding portion of the genome in S. gregaria makes the assembly more challenging. Can the authors please expand on this argument?

 2. Was any kind of filtering done to remove microbial contamination? The animal material protocol in the supplementary methods does not mention starving the insects before DNA extraction. Can this have introduced microbial contamination from the feed even though the leaves were washed?

 3. This manuscript represents a mammoth amount of work that has gone into the genome assembly. The standard of quality for a genome assembly has increased vastly since the L. migratiria genome was published in 2014. Since sourcing high quality DNA from the insects does not seem to be a major challenge as far as I know, were long range scaffolding methods like Hi-C or BioNano explored for chromosomal scale scaffolding?

Is the work clearly and accurately presented and does it cite the current literature?

Yes

If applicable, is the statistical analysis and its interpretation appropriate?

Not applicable

Are all the source data underlying the results available to ensure full reproducibility?

Yes

Is the study design appropriate and is the work technically sound?

Yes

Are the conclusions drawn adequately supported by the results?

Yes

Are sufficient details of methods and analysis provided to allow replication by others?

Yes

Reviewer Expertise:

Arthropod genomics and transcriptomics, Comparative genomics and Metagenomics

I confirm that I have read this submission and believe that I have an appropriate level of expertise to confirm that it is of an acceptable scientific standard, however I have significant reservations, as outlined above.

## References

[1] : Locust Bacterial Symbionts: An Update. *Insects* .2020;11(10) : 10.3390/insects11100655 10.3390/insects11100655 32987763PMC7598710

# Response

**Published:** 2021-05-06

**Authors:** Heleen Verlinden

**Affiliations:** Catholic University of Leuven, Belgium

We would like to thank the reviewer for this positive feedback, as well as for recognising the challenges we have faced. Since sequencing technologies are evolving very fast, we agree that future work will further improve the current genome assembly. Therefore, we designated this as the “first genome assembly”. In addition, with the release of our genome data, we are convinced that many additional analyses are becoming possible. Locusts have always been -and still are- research models for studying a wide range of research questions in different areas of interest. With this manuscript, we therefore chose not to focus on a very specific research question, but communicate the main results obtained of our genome sequencing effort. Moreover, given the current locust situation, we felt that it was important to make available this first genome draft to the international scientific community and to stimulate readers -worldwide- to address their specific research question(s).

 1) We agree that these analyses will be of great value and sincerely hope to include these in future research, however they are out of scope of this first draft publication.

 2)We took many precautions to reduce the risk of contamination. The locust cages were meticulously cleaned multiple times a week, all material that entered the facility was either new or sterilised or both. Food was bred separately and washed carefully. Gloves, lab coats, face masks were worn upon entering the rooms. Our animals all looked healthy, with no signs of infections.

 During the dissections, we were extremely careful not to damage the gut and did not include any digestive tissues. We checked for contamination by BLASTX and BLASTN with sequence databases from a number of vertebrate, plant, fungi, gregarina, amoeba, bacteria and protoctista and could not find any significant contamination. We therefore did not perform any form of filtering for endosymbiont/microbial contamination.

 Following sentences have now been adapted/added to the supplementary materials and methods to make this clearer for the reader:

 “The entire breeding facility was meticulously cleaned multiple times a week throughout the entire breeding period to avoid any form of contamination. All materials that entered the facility were either new or sterilised or even both. Food was bred separately and washed carefully. Gloves, lab coats and face masks were worn upon entering the facility.

 The locusts showed no external signs of disease or contamination, displayed clear phenotypic characteristics of solitarious locusts and underwent the sixth larval stage characteristic of the solitarious phase (Pener and Simpson, 2009; Cullen et al., 2017). The animals were very carefully dissected and specific attention was paid to avoid any contamination with gut material.”

 “We checked for contamination by BLASTX and BLASTN with sequence databases from a number of vertebrate, plant, fungi, gregarina, amoeba, bacteria and protoctista and could not find any contamination. We therefore did not perform any form of filtering for endosymbiont/microbial contamination.”

 Minor points:

 1. In general, a bigger genome takes more reads and thus also more budget to obtain enough sequencing coverage. Although we did not concretely know how expanded the non-coding portion of the genome would be, we had expected the presence of many repetitive regions, which can indeed make assembly more complicated and challenging. We therefore anticipated to this by including a long-read technology like PacBio, to improve the assembly process of short Illumina reads. We specified that we also expected more repetitive regions and added two references here:

 Dominguez Del Angel, V., Hjerde, E., Sterck, L., et al.: Ten steps to get started in Genome Assembly and Annotation [version 1; peer review: 2 approved]. F1000Research 2018, 7(ELIXIR), 148.

 Tørresen, O.K., Star, B., Mier, P., et al.: Tandem repeats lead to sequence assembly errors and impose multi-level challenges for genome and protein databases. Nucleic Acids Res 2019; 47(21), 10994–11006.

 2. As clarified above, we took many precautions to avoid contaminations and indeed checked if there was contamination. Since no significant contamination was observed, we did not perform any form of filtering.

 3. We performed PacBio sequencing, in addition to the Illumina sequencing, to obtain long reads.  We agree that newer technologies may be very useful to further optimise the genome sequence assembly.

# Peer review: Reviewer response for version 1

**DOI:** 10.5256/f1000research.27753.r71637

**Published:** 2020-10-05

**Authors:** Uwe Homberg

**Affiliations:** 1 Animal Physiology, Department of Biology, Center for Mind, Brain and Behavior (CMBB), University of Marburg, Giessen, Germany

This is a marvellous paper based on an enormous effort for genome assembly in this insect. The work is urgently needed in order to promote a large number of studies on the behavior and physiology of this insect. The data are highly likely to ultimately better understand migratory behavior in the desert locust as well as its phase polyethism. I have no comments or suggestions for further improvements of this already excellent achievement.

Is the work clearly and accurately presented and does it cite the current literature?

Yes

If applicable, is the statistical analysis and its interpretation appropriate?

Yes

Are all the source data underlying the results available to ensure full reproducibility?

Yes

Is the study design appropriate and is the work technically sound?

Yes

Are the conclusions drawn adequately supported by the results?

Yes

Are sufficient details of methods and analysis provided to allow replication by others?

Yes

Reviewer Expertise:

neurobiology of the desert locust, neuropeptide research in desert locust, neuroanatomy of the locust brain

I confirm that I have read this submission and believe that I have an appropriate level of expertise to confirm that it is of an acceptable scientific standard.

# Response

**Published:** 2021-05-06

**Authors:** Heleen Verlinden

**Affiliations:** Catholic University of Leuven, Belgium

The authors would like to thank the reviewer for this very positive feedback on their manuscript.
